# Book Reviews

**Published:** 1810-12

**Authors:** 


### CRITICAL ANALYSIS

OF

### RECENT PUBLICATIONS

IN THE

### DIFFERENT BRANCHES OF PHYSIC, SURGERY, AND MEDICAL PHILOSOPHY,

---

*An Address delivered to the Lincolnshire Benevolent Medical Society at their anniversary Meeting in* 1809. *Containing an account of the proceedings lately adopted to improve Medical Science, and an Exposition of the intended Act for regulating Medical Education and Practice. To which is added an Appendix, comprising the Acts of Henry VIII. and the Correspondence had with the public bodies; together with the legal opinion of an eminent Counsel on the subject of Medical regulation, &c.* By Edward Harrison, M. D. F. R. A. S. Ed. &c. &c. 8vo. Bickerstaff. London, 1810.

Undismayed by the want of success which has hitherto attended his exertions, Dr. Harrison, though in some respects he has abandoned his original plan of extending the term of education, continues to stand forth the champion of Medical Reform. The volume before us contains a full account of the proceedings which have yet occurred on the subject. The transactions of the meetings at which Sir Joseph Banks presided have already been made public, and in a late Number ber of this Journal, Dr. Harrison's Memorial was inserted. We shall therefore, at present, merely state the substance of the Bill intended to be laid before the ensuing Session of Parliament, and offer some observations upon the general question.——Every person who shall practise for gain, in Great Britain or Ireland, as a physician, surgeon, midwife, apothecary, or veterinary practitioner; or who shall sell medicines or drugs, or chemical preparations by retail as an apothecary, compounder, or vender of medicines or drugs, shall cause to be entered in a register, his name, place of abode, and the capacity in which he practises, or vends medicines, drugs, &c.—Upon changing his place of abode, or the capacity in which he practises, he shall be required to make a new entry thereof, within the space of one month after such change. At the time of making the entry, the person is to receive a certificate of the capacity in which he is registered to practise, &c. Physicians, Surgeons, Midwives, Apothecaries, &c. &c. are required, under a penalty, annually to take out a certificate, that they have been duly registered.—They are also obliged, under a penalty, to put upon, or over their doors, the capacities in which they practise. The qualifications for enabling them to practise according to this Bill, are as follow :—a Physician must have taken the degree of a Doctor or Bachelor of Physic, in some University, and must have obtained "a diploma or authority to practise as a Physician, from the Royal College of Physicians of London, if he reside in England, or Wales; from the Royal College of Physicians of Dublin, if he reside in Ireland; and from the Royal College of Physicians of Edinburgh, if he reside in any other part of Scotland than in the counties of Lanark, Renfrew, Dumbarton, and Air; and if he reside in any of these counties, then from the Faculty of Physicians and Surgeons of Glasgow. A Surgeon must obtain a diploma or authority to practise, from the Royal College of Surgeons of London, if he reside in England or Wales; if in Ireland, from the Royal College of Surgeons of Dublin; if in Scotland, from the Royal College of Surgeons of Edinburgh, except he reside in the counties of Lanark, Renfrew, &c. then from the Faculty of Physicians and Surgeons of Glasgow.

No person acting as apothecary, vender of medicines, &c. shall be entitled to be registered to act as such, until he shall have served an apprenticeship, by regular indentures, to a Physician or Surgeon, who dispenses medicines, or has the care of an hospital or dispensary; or to an Apothecary; nor until he shall have been examined by, and shall have obtained a certificate from the Company of Apothecaries in London, or Dublin, or in Edinburgh, from a Medical Board to be esta-

 blished blished by the Commissioners appointed to carry this Act into execution, and if he reside more than seven miles from such Company or Board, then either from the aforesaid Company or Board, or from Medical persons appointed by the said Commissioners for that purpose.

No person shall be entitled to be registered to act as a Man-midwife, for gain, unless he be a Physician, Surgeon, or Apothecary, and have obtained a Certificate from a Board of Midwifery Practitioners, to be appointed by the said Commissioners. Female Midwifes shall not be registered to act as such, until they have been examined and licensed by a Board of Midwifery Practitioners. Farriers and Veterinary Practitioners are to be examined, and to obtain authority to practise as such, from some person appointed by the said Commissioners, before they can be entitled to be registered to act in their professional capacity.

Bone-setters will be allowed to act and practise under Licences from Justices at Quarter Sessions, who shall have the power of withdrawing any such Licence whenever they shall think proper.

"Every person who shall not have entered into practice before the passing of this Act, as a Physician, Surgeon, Man-midwife, Apothecary, Compounder, or Vender of Drugs by retail, shall pay to the Registrar, when his name is first entered upon the register, the sum of twenty pounds, to be applied to carry into execution the purposes of this Act."

The Bill does not extend to prevent persons from selling drugs or medicines subject to a stamp duty.

The remainder of the Bill relates to the appointment of Commissioners, the regulation of their duty, and the application of the monies received.

From the whole of his conduct in this affair, we are disposed to believe, that Dr. Harrison has acted from good motives, and is convinced of the utility and practicability of the object which he has in view. We confess, however, that we observed, with compassion, his former attempts to induce the Royal College of Physicians to co-operate with his party, because we well knew, that the attempt would be abortive; it is the interest of the College, according to its present constitution, to tolerate an inferior description of Practitioners, in order to secure an ascendancy for its own fellows. Dr. Harrison satisfactorily proved, what no intelligent and impartial man will deny, that great abuses prevail in our profession throughout every part of the empire; in Lincolnshire, and we doubt not the same may be the case in many other counties, he ascertained that empirical pretenders exceeded the regular Faculty, in the proportion of nine to one, and that many persons sons practised as Physicians and Surgeons, who had no title or claim to those honourable appellations. Sir Lucas Pepys, the President of the College, admitted that these abuses existed, and that it was expedient that they should be removed. The Royal College even roused itself from its long slumber, published advertisements in the newspapers, sent addresses to the Quarter-Sessions, and circulated lists of its members, "in order that the names of all those may be known, who, having been examined by the College according to law, have been deemed competent to practise as Physicians."— These addresses produced about as much effect on the honest justices, as the bulls of the Pope upon Dissenters; we did not hear of a single delinquent being punished or interdicted. In fact, we believe that the College discovered that its power did not extend ten miles from Warwick-lane, and was unwilling to expose its limited authority by promoting an inquiry into abuses which it could not prevent.

Finding that he could not obtain assistance from any of the incorporated Bodies of Medicine, and Surgery, in London, or Edinburgh; Dr. Harrison projected the Bill, the substance of which we have just stated. We do not, however, anticipate a favourable result from such an application to Parliament, and much doubt, that were the plan carried into execution, its effects would be salutary. The College of Physicians was instituted by Royal Authority, and from the nature of its constitution is intimately connected with our two English Universities, and consequently may be regarded as forming part of the aristocratic establishment of the country: any attempt at reform, therefore, not sanctioned by the College itself, must be hopeless, whilst these institutions continue to be upheld by government.

Can we even for a moment suppose that our legislators deem the health of the people of any importance, when a large revenue is derived from the distillation of ardent spirits, and the sale of empirical nostrums, which we apprehend are infinitely more destructive than the combined efforts of all the persons practising medicine without being duly qualified, in the three kingdoms?

Dr. Beddoes, in his letter to Sir Joseph Banks, observes, The association must well know, that perjury is constantly kept up by the means taken to recommend quack medicines; that in consequence of money expended in their purchase, families above the poorest class are frequently deprived of the necessaries of life: that there are instances where people have sold the bed from under them, (the rage for quack-medicines, in some familiar instances, being just like the habit of dram-drinking,) and that the resort to these preparations is far from being only the natural catching at a straw, in him who feels himself drowning. The prostitute stitute is not content to wait at home in readiness to satiate already excited appetites, but roams abroad to practise all the arts of provocation. Neither is the quack content with the spontaneous yearnings of credulity; his lures are spread in all our streets. The office of every country newspaper is an office for his medicines, and often for nothing else. From the sale, the printer procures advertisements, and pays himself. Every country bookseller almost is stimulated by an enormous commission to pass them off. I think, therefore, that a scheme for a reform of medicine, without the abolition of quack-medicines, is almost as hopeless, as one for making the rattle-snake harmless, leaving the venom fangs in his jaws; nor should I be surprized, if such a scheme pass, to hear of a grand dinner among the quacks, and each reformer being toasted in a bumper."

These real grievances, grievances at which our nature shudders, are suffered to remain; whilst the proposed Bill is calculated in its operation to subject a respectable, and in general, a well-educated body of men to very irksome restrictions. Is it not enough that after having taken out a diploma in a regular way, a Physician again submits to be examined by the Censors of the College, and pays a large sum, of the application of which he is kept wholly ignorant; but that he must, after all this, be registered, pay twenty guineas, and annually take out a certificate; in short be subjected to the same regulations as are to be imposed on the most inferior practitioners of the art! It is true that men of ability and address, without the advantages of scholastic education, or of College discipline, will obtain the confidence and the patronage of the public in an eminent degree, but we cannot altogether see the justice of attempting to deprive such men of doing good to others, and benefiting themselves, because some of them may do mischief. If a Physician with the advantages of a liberal education, and a regular diploma, cannot make his superiority be acknowledged, we must confess that we do not see the propriety of protecting him, and imposing his dulness on the public, by restrictions which would exclude men of talent, who may know how to cure a disease, though they cannot construe Hippocrates.

We presume Dr. Harrison has perused an ancient treatise, entitled "A Corner-stone laid towards the building of a New College, (that is to say, a new body of Physicians), &c." If so, he will probably recollect the following passage. "It seems to be one of the most unreasonable things in the world, and nothing can be more destructive to the liberty of the subject, or make a man more miserable, than that if he be sick, he should not with freedom use what Physician he believes can best cure him, but he must be limited to an accepting of such such or such an one, of such a company, or else can have none that he fancies, but the man shall be molested, perhaps undone, for doing the sick service ; and all under a supposal of avoiding thereby the use of bad physick among the people."

The emoluments of a medical man must depend on the opinion of the public. If illiterate and uneducated practitioners bear down those of an opposite description, we must conclude that the public is very ignorant, and that the present race of Physicians is too learned for them, and must consequently seek some other means of support, or repair to other countries where medical learning is required. But we have no hesitation in asserting that this is not the case ; the times are unfavourable to mediocrity in medical attainments ; chicane and intrigue may impose for a time, but soon give place to real merit. If many men of indisputable worth never acquire much practice, such persons will generally be found pursuing other objects than those of their profession, or they labour under disadvantages which their genius cannot overcome.

The same objection to the Bill will hold with regard to Surgeons: whenever the field of practice is extensive we may depend on finding adequate Surgeons; if people prefer dwelling in villages and obscure situations, they must be content with what chirurgical assistance they can obtain ; no College, no Parliamentary edict, will ensure the lord of a solitary mansion the aid of a skilful Surgeon, unless the neighbourhood be populous. As to Apothecaries, we believe, that throughout the kingdom, their education, skill, and good conduct, is quite equal to the drudgery which they endure, and the moderate stipend they receive. Would men of university education, and with scientific acquirements submit to the daily labour of a country apothecary ? Dr. Harrison has, to be sure, adduced some lamentable instances of the evils occasioned by *soi-disant* medical practitioners, but from the very circumstance of these cases being shocking to humanity, we contend that the danger of their increasing or being tolerated is diminished. The Bill, if enacted, would deprive many districts of regular practitioners, because their emoluments would not be adequate to the irksome and expensive regulations imposed on them. In fine, we have nothing to praise in the work, but the intention of the author, who we regret has bestowed so much time and attention upon this stubborn subject. It appears to us that he has attempted to lop some mishapen branches of an old tree, whilst he feared to apply the axe to the decayed trunk. One of his suggestions indeed merits merits consideration, and we hope, whatever be the fate of his Bill, that the scheme may obtain the consideration which it deserves. It is a notorious and distressing fact, that in the first city of the world, the seat of a Royal College of Physicians, there should be no complete and liberal system of medical instruction. Our hospitals afford the most extensive scope for practical observation, and scientific investigation; our lecturers are well versed in their art, but disturbed by the engagements of private practice, they have little leisure for systematic arrangement, or very profound attainments, whilst teachers of less eminence, cannot command a number of pupils sufficient to remunerate them for their toil. The knowledge of medicine cannot be obtained at Oxford and Cambridge; and in the Edinburgh school, the confined opportunities of acquiring practical information, are hardly compensated by the attention which is bestowed on theoretical and erudite disquisition. Dr. Harrison then proposes the establishment of a Medical School in London, in which he was preceded by Dr. Beddoes,* with whose acute and sensible remarks on the subject, we shall conclude this article.

I trust, (he observes) if it ever come to law-making, that the objection will be radically removed, by endowing London, at once, with the privilege enjoyed by every great metropolis in Europe besides, and particularly suited to a great metropolis. An attempt at improvement, short of this, though it may not leave British medicine as ineffective as the pamphlet of Dr. Harrison found it, will leave it much more ineffective than the means of instruction allow, and the public good requires. Why, in the name of common sense, should not London be authorized to call candidates in our faculty to the bed-side, as it does those in another to the bar? Neither English university would, I dare say, view

---

* Dr. Beddoes might have obtained the first suggestion of this plan from the "Corner-Stone" before quoted. The author of that rare tract, Dr. Huyberts, observes, "If the whole body of Physicians here in this city, be really the Physicians of London, why may they not, being part of the city, be taken hereafter under the city government? Be obliged to take apprentices, such young men as have taken degrees in other arts at some University; who, when they have served their time at work under a city Physician, may then be made a free Practiser of *London*. Such a populous city is the only place (being a theatre of all diseases) wherein to breed up men Physicians indeed; such as may practise with real knowledge; not fill the world with cobwebs of idle speculations and notions, as men of the old way of education are wont to do; and which may furnish his Majesty's armies and fleets royal with Physicians, as the Societyof Chirurgie do with Chirurgians; and be content to submit to a law, that if they *run away from the city in the time of plague*†, or depart without special licence of authority, to forfeit their freedom of practice therein any more."

† It seems that upon the plague breaking out in London, the city was abandoned by most of the fellows of the college, Dr. Wharton, and three or four more only remaining.

the the measure with jealousy. The members, and the whole public must perceive, that London is the spot in Great Britain, and probably in the whole world, where medicine may be taught, as well as cultivated, to most advantage. Nor would it be easy to estimate the number of persons, whom our government has suffered to linger in misery, or to die in the flower of their days, simply from having so long neglected to found a complete Medical Seminary there The claim is on the part of human misery, not of national pride. By the force of its unrivalled opportunity alone, with but few teachers, either celebrated, or deserving celebrity, though they now seem to have grown as thick as haberdashers, London has, perhaps, more than equalled the most celebrated Seminaries, and it would, I think, amount to a sort of treason against the common-weal, if they should remain without being seconded by the whole public power, after the state of medicine comes to be a matter of legislative deliberation. In the course of many years, I not only never heard a specious objection, but generally found, when the leading ideas were propounded, that every hearer followed them up to the same conclusion.

But let us not, at least not wilfully, take a station below Paris. Let our regulations aim at still greater maturity in those whom we shall send forth to practise, and teach the healing art *ubique gentium*. What would it avail to increase the breed of Practitioners, akin to those coffee-house politicians of ours, to whom the leading paragraph of the Morning Post stands in the place of history, and political economy; practitioners, all absorbed in the last curious case at Guy's, or the last curious opinion from St. Bartholomew's; and not much more deeply skilled in the disorders of the individual, than the news-mongers are in those of the body politic.

Every well wisher to medicine, and to mankind, would desire to see Edinburgh maintain or recover what she ever had of real excellence.—The contest would be generous if fairly carried on, and the competitors be allowed the fullest exercise of their faculties. But let not London have her hands tied behind her, that the sons of Edinburgh may have the delight of beholding her superiority, or at least escape being mortified by her manifest inferiority."

---

*Description of an affection on the Tibia induced by Fever, with Observations on the Treatment of this Complaint. By* Thomas Whateley, Member of the Royal College of Surgeons in London. 8vo. London. 1810. pp. 59. Coloured plate. Callow.

The complaint which this essay professes to describe, is a disease of the Tibia. It is pointed out by one or more small openings in the integuments lying upon the surface of this bone, through which an instrument may be passed into its cavity. A proper examination with the probe will detect one, or sometimes several loose pieces of bone lying in this cavity; cavity; and which being larger than the external opening, are necessarily confined within the Tibia, and being dead matter, certainly and invariably prevent the healing of the ulcer.

The curative process is simple and effectual. The external opening must be enlarged to a dimension that admits of the exfoliated portion of bone being removed; and then with easy dressings, a proper attention being given to the general health, and care taken to avoid the causes of irritation, the ulcer soon heals.

The only question seems to be how this opening into the cavity of the Tibia is to be enlarged, for the purpose of extricating the portion of dead bone. Most surgeons would use the trephine; Mr. Whateley, however, prefers caustic. (*Kali purum.*)

This disease, which is undoubtedly an internal exfoliation of the Tibia, is, perhaps, generally a *sequela* of fever; or, is some way connected with, or excited by, a preceding febrile affection.

"Having observed," says Mr. Whately, "that in almost all the cases of this kind, which I have had in hand, the complaint had been preceded by fever, I have been led to consider the dissase in question as a febrile affection of the part. I should inform the reader, that the case, now before him, is not one which frequently occurs. I do not recollect having, in the course of my practice, had more than about thirty of them entrusted to me. But having, in the course of many years, observed a certain peculiarity in the disease, I took notes of most of the cases, which came under my notice, conceiving that I might, at some future time, be thereby enabled to afford useful information to the public, on the treatment of this complaint.

"These cases occur more frequently at the middle period of life, than at any other.* In most instances, that came within my observation, the preceding fever was of considerable duration; and sometimes so violent, during its continuance, as to confine the patient to his bed, and often to be attended with delirium. Some time after the patient's recovery, he was seized with pain in one of his legs. The period at which this pain commences, is not the same in every case. In some patients, it takes place immediately on the termination of the fever; in others, it is not

* I took notes of twenty-two cases which have been under my own care, the ages of these are as follow;

| | | |
|---|---|---|
| Of the age of 14 | - - | 1 |
| ——— 18 | - - | 1 |
| ——— 19 | - - | 1 |
| Between 20 and 30 | - - | 7 |
| ——— 30 and 40 | - - | 11 |
| ——— 40 and 50 | - - | 1 |
| | | 22 |

experienced experienced till a few days afterwards; in others again, not till several weeks, and in others, not till many months have elapsed. Yet I should observe, that I have always found the disease to make its attack, within twelve months from the termination of the fever."

The phenomena and symptoms of the complaint are described with minuteness and precision.

"In most of the cases, the pain in the leg was acute, and appeared to the patient to proceed from the interior of the Tibia. The pain, indeed, was not equally violent in every case; as some of the patients were confined to their bed for several days, or weeks, and deprived of sleep for a considerable time; while others were able to go about. No external swelling or inflammation is, in general, to be expected, immediately on the commencement of pain. Yet these appearances are seldom long in following; they are usually visible within a week, and sometimes in two or three days. In some cases, a general inflammation and swelling over the whole limb first appeared, and soon after a more circumscribed inflammation, near the affected part of the Tibia, in which a fluctuation of pus might be felt. In other cases, the inflammation and swelling never extended to the entire limb, but were, with the subsequent formation of pus, confined to a small circle on the Tibia. In most instances, the suppuration was confined to one part of the Tibia; and this was often about the middle of the leg, and the centre of the bone. In many cases, however, the suppuration took place in other parts of this bone; namely, within a few inches of the instep, a little below the knee, or in some of the intermediate spaces. Sometimes it has happened, that two or more distinct suppurations, and as many separate openings into the cavity of the bone have taken place. These were sometimes found within an inch or two of each other; at other times further apart. There was some variation likewise, as to the time of their appearance: some weeks elapsing between the commencement of each suppuration, in some cases, while in others, all appeared nearly at the same time.

"Although a fluctuation of pus might be discovered, within a short time after the disease had taken place, the matter did not usually burst through the skin as hastily as it does in some other suppurations. This discharge, however, sometimes took place within a week or two, from the first appearance of matter; but in many cases, the pus continued in a confined state, for many months, and required the use of the lancet to set it free.*

"In most of the cases, the violence of the pain ceased: the inflammation and swelling abated, as soon as the fluctuation of matter was felt; and in a short time afterwards, the patient was generally able to walk about. Soon after the discharge of matter, the wound usually contracted to so small a size, as scarcely to admit the point of a probe; and a small quantity of pus continued to ooze from the aperture. In

* In the greater number of cases, the suppuration is small, often containing not more than a tea-spoonful or two of pus. In other cases, however, I have seen a larger suppuration, in which, an ounce or more, of matter is formed.

some cases, however, the ulcers closed up for a few days, and then burst out again.—If in this state of the disease, a probe is passed carefully into the little orifice of the ulcer, it usually enters readily, into the cavity of the Tibia,* where the loose piece or pieces of exfoliated bone are frequently to be felt. Under these circumstances, I have never known a perfect cure to take place, unless where the appropriate treatment had been resorted to.

"In this disease, the surface of the Tibia, immediately around the orifice, becomes often, though not always, knotty and irregular, and the periosteum is sometimes thickened, so as to give an appearance, resembling that of a venereal node. This disease, however, differs totally from those affections of the Tibia produced by syphilis, as it does likewise from those usually denominated necrosis, or from any of those which are produced by scrophula. In short, as far as I have been able to observe, it is a disease sui generis, the relic of fever usually affecting the Tibia.†

"From the violence of the pain, and sudden erosion of the bone, an inflammation appears to attack its internal part. Suppuration takes place, and matter is first formed there, and then makes its way through the substance of the bone, to the outer integuments.—As, however, the ulcerative process in the bone sometimes takes place in different parts, thereby producing several apertures in the Tibia, matter is, in these cases probably, formed in distinct places, previous to the ulcerations in the bone. One of the most remarkable circumstances attending these cases is, that one or more pieces of loose bone are usually found within the cavity of the Tibia, opposite to each aperture.

"Some of these exfoliations are so small and thin, as to require a nice examination with the probe, in order to discover them. They are, however, generally found of a larger size, sometimes even exceeding an inch in length. I have always found these exfoliations of an oblong and spiral shape, and evidently separated from the internal laminæ of the Tibia within the cavity of the bone. The separation of such a portion of bone is probably the effect of the previous inflammation and ulceration; by which it is deprived of circulation, and of course loses its connection with the living bone, of which it was once a part."

Having enquired into the probable cause of this disease, and passed through the various appearances it assumes, the author proceeds to describe the curative process.

"During the painful and inflammatory state of the disease, and before the erosion of the bone has taken place, it would be proper to apply emollient fomentations and poultices to the limb: to keep the patient in bed; and to endeavour to ease the pain, by occasional doses of opium. I must confess, however, that I have seldom seen the disease in this stage. I have commonly been applied to, after the erosion of

---

* In one or two cases, I have perceived this orifice so small, as not to admit the round end of a probe, of the usual size. In a few other cases, it has been so oblique, as to be entered with much difficulty by that instrument.

† I have some recollection of having, many years ago, seen this disease in the Fibula; but in that case, the Tibia was likewise affected.

the the bone has taken place, and when the patient has been, in some measure, able to follow his usual employment. In this stage of the disease the cure of the ulcer, by the common external remedies, is impossible, the loose piece or pieces of bone, pent up within the cavity of the bone, must set that mode of procedure at defiance. The imprisoned exfoliations, therefore, must be extracted, and this is desirable to be done, by the mildest methods possible; avoiding all unnecessary pain and operations to the patient.

"The loose bone within the cancelli, is commonly longer than the ulcerated aperture in the bone. It is in many cases, indeed, considerably longer, and though confined to its situation, it may sometimes be taken hold of by a pair of forceps, and moved upwards and downwards, in its bony case. The examination by the probe should, therefore, be made with great care and gentleness; otherwise, much unnecessary pain will be excited. It may happen also, that the exfoliated bone may be removed from the favourable situation it is in, when it is directly under the ulcerated aperture, and be pushed under the arch of the Tibia, as I have more than once experienced; by which the difficulty of extracting it must be greatly increased.

"As the orifice in the Tibia, as well as the exfoliations, are of different sizes in different cases, and as these are generally covered by the integuments and granulations, it requires a nice examination with the probe, to ascertain the true state of the parts. In some cases, as before observed, the aperture in the Tibia is so small, as not to admit the round end of the probe, to pass into the cavity of the bone, and the contrary end, or an eyed probe, will pass into it with so much difficulty, that no accurate examination of the parts beneath can be made. In most cases, however, it freely admits the round point of a probe; and our attention should first be directed, to find out the size of the aperture in the bone. Having ascertained this, we should next examine, whether there be a loose piece of bone within the cancelli. The size of the aperture may be known, by passing the probe, in a gentle manner, from side to side in all directions. But it is not so easy, either to find out the exfoliated piece, or to ascertain its exact dimensions, when discovered.

"Where the aperture in the Tibia is large, and the exfoliated piece very small, the latter may be moved about, with the end of the probe, so readily, as to leave no doubt of its easy extraction, by the introduction of a pair of fine forceps. Under such circumstances, the operation should be instantly performed; and if the whole be removed, the wound will generally heal up in the course of a few weeks: the application of any common dressing, aided by bandage, will be sufficient for the purpose. The utility, therefore, of first examining with great nicety, by the probe, in order to avoid, if possible, the use of more violent remedies, must be evident.

"In more difficult cases, the exfoliated piece may, likewise, be readily moved in the cavity of the bone, by the end of the probe; yet the hand of the operator will be sensible that it is confined, by the sides of the surrounding Tibia. In some cases of this kind, the opening in the Tibia may be large enough to admit of our taking hold of the exfoliation, by a pair of common forceps; but in others, it is so small, that a a pair of the finest forceps that can be made, cannot be made to enter, so as to embrace the bone. When possible, we should endeavour to extract the exfoliated bone by the forceps; and in these attempts I have readily succeeded, when the exfoliation was not longer than the aperture in the Tibia. But where it proved to be of greater length, which might be known, by its being moved upwards and downwards in its bony case, by the forceps, but whence it was not possible to extract it; I have been sometimes able to accomplish the extraction, by moving it upwards or downwards, as far as it would go, and then raising one end of it. Where I could not succeed by this procedure, I have been sometimes obliged to resort to a more violent method, that of endeavouring to break the exfoliation into two parts, by means of the forceps. This I have been able to do, where it has not been very strong, and having thereby extracted it, the cure has been speedily completed.

" There are other cases, however, in which methods, very different from those, must be taken. We may have the strongest reasons for concluding, that there is a loose exfoliation, within the cancelli, and yet may not be able to feel it by the probe. In this case, as well as when the opening in the Tibia is too small, to admit an exfoliation to pass, by any of the methods above described, we must apply the kali purum to the integuments around the ulcerated opening in such a manner, and in such quantity, as to destroy them, to the extent of about half an inch from the centre of the opening. This is done with a view to expose as much of the surface of the Tibia, as is requisite for the cure.* The proceeding, here recommended, is attended of course with some pain; and it requires particular attention, in order to prevent the kali from destroying more of the integuments than is necessary; and from penetrating through the orifice into the cancelli.

" This caustic may be applied in several different ways. That which I prefer is, to take about as much of it (bruised into parts of the size of a pin's head) as will lie upon a seven shilling piece, and apply it, both to the ulcerated opening in the skin, and to the surrounding integuments, to the extent already mentioned. Pieces of dry lint, or adhesive plaister, should be applied around the caustic, to prevent it from extending on the adjoining skin. In a few minutes, the kali liquifies, and begins to operate.

" The leg should be placed in a horizontal position; and if the caustic appear to act equally, the limb must remain in that position for about ten minutes, or a quarter of an hour; after which, a pledget of dry lint should be applied over the kali, large enough to cover the adjoining sound skin. The leg should then be slightly bandaged with a flannel roller.

" It is to be observed here, however, that during the action of the caustic, some additional attentions are not unfrequently required, in order to ascertain the depth to which it has penetrated. If in one part, the integument is thicker than in another, or if the caustic is acting more

* The orifice in the bone is situate so near to an angle of the Tibia, in some cases, that it does not admit of its being exposed, to the extent of half an inch, on all sides of it.

powerfully powerfully on one part than on another, a little of it may be removed with the probe, to or from such part; or a little more fresh caustic may be applied to any particular part, if it appear to require it. During the action of the caustic, particular attention must be paid to its effect on the little ulcer on the skin, and on the orifice in the Tibia, as it is desirable to prevent its penetrating the cancelli, much harm and disturbance being likely to arise from its action on this part.

"If the integuments adjoining to the wound be thick, the kali may be applied to this part, nearly in the same quantity as to the other parts; as, in general, it ceases to act, before it can penetrate into the cancelli. But if the integument, on this part, be very thin; or if there be no integument or granulation over the ulcerated orifice, a small piece of lint should be passed down it to the cancelli, previous to the application of the kali, to prevent it penetrating too far: a piece about the size of a pea will generally be sufficient. This should be rolled with the finger and thumb, and firmly pressed down the orifice by a probe, till it passes into the cancelli; where it should remain. If this should not perfectly plug it up, a second, or even a third pledget should be applied. This is often attended with some pain, from the pressure made on the granulated flesh, at the bottom of the wound, which generally springs from within the cancelli, and is exquisitely sensible. The kali should remain on the part to which it is applied, for about six hours. After this, the wound should be dressed twice a day, with some dry lint, and an emollient poultice. In about a week, or ten days, from the application of the caustic, the slough will come away.

"If the caustic have performed its office, the surface of the Tibia will be perfectly bare; and now the unequal, knotty state of the bone, and thickening of the periosteum, will often be seen; and if the granulations on the surface of the orifice have been destroyed by it, the loose exfoliation within the Tibia will often be visible to the naked eye. But where the granulations are not destroyed, the internal exfoliation cannot be discovered without introducing the probe into the wound, as before directed.

"Sometimes the surface of the Tibia around the ulcerated aperture, will be found, on the separation of the slough, not to be sufficiently exposed.—In this case, a little of the kali must be applied a second time, to such parts as require it. This should be done in the course of a few days, otherwise the wound will begin to fill up with granulations. Though a repetition of the caustic is seldom attended with as much pain as accompanies the first application, it being seldom necessary to destroy a fresh portion of the skin; it should nevertheless be applied with all the precautions, recommended in the first instance; the like dangers being to be guarded against, on the subsequent application as on the first. It must be remembered, however, that a proper quantity should be applied, otherwise the surface of the bone will not be sufficiently exposed, when the new slough separates. When the second application has been properly conducted, we seldom have occasion for a third.

"The surface of the Tibia, with the aperture into its cavity, being thus exposed to view, we proceed to search for the loose exfoliation, if it be not already visible. The orifice in the Tibia must again be carefully fully examined by a probe, and as this examination can now be assisted with the eye, the size and situation of the exfoliated piece will for the most part be readily discovered. If indeed the granulations within the cavity of the bone shall not have been destroyed by the caustic, some impediment to the examination may arise from them, as they are in general extremely sensible, and are apt to bleed, on the slightest touch of the probe. But as the exfoliated piece generally lies buried in these granulations, and is perfectly detached from the contiguous bone, it may frequently be discovered, by its yielding to the pressure of the probe.

"Its situation being discovered, and its size, compared with that of the aperture in the Tibia, pretty fairly ascertained, we must endeavour to extract it by the forceps; and sometimes we shall succeed, where the endeavours made, previous to the application of the caustic, have failed. The exfoliated bone, however, will often be found to be larger than the opening in the Tibia, in which case it will, in general, be impossible to extract it, till the orifice has been widened.

"If the trephine be used for this purpose, it may be sometimes difficult, though probably never impracticable, to remove the piece encircled by the instrument after it has been worked to a proper depth. I remember to have succeeded many years ago by this method, in extracting a large piece of bone, pent up within the cancelli. The case to which I allude occurred to me when I was a very young practitioner, and has been already published. From the date of this operation, there has not occurred a single case in my practice, in which I have found it necessary to use the trephine. I have, for some years, practiced with great success a method of enlarging the opening in the Tibia, which is much more simple, as well as less painful. It is by means of the kali purum; and the way in which I use it is as follows: I apply this caustic to the bare surface of the Tibia, around the hole, and to the sides of the hole itself, after the separation of the slough; taking care to guard against its touching the adjoining parts, or penetrating into the cavity of the Tibia: each of which dangers may be prevented, by the application of the lint, as before directed. In about ten minutes after the application of the kali, the bone should be covered with a pledget of lint; and on the following day the kali should be again applied to all the parts, as before. After this the limb should be dressed twice every day with a fresh poultice; it should be lightly covered with a flannel roller, and the patient may be generally permitted to take moderate exercise. In a month or less from this period, an exfoliation of all that part of the bone, to which the caustic has been applied, will take place.* This effect may be known by occasionally pressing a probe upon the surface of the bone. For when the intended exfoliation is accomplished, the ex-

* I have not known this exfoliation take place in less than three weeks from the application of the caustic, and it happens, not unfrequently, that it is not separated in less time than a month. The granulated flesh around it, therefore, generally covers this dead part of the bone before it is taken out. This, of course, obscures it from the view, but when loose, it is as readily extracted, with the loss only of a few drops of blood, as if it was perfectly exposed.

foliated foliated piece gives way, on the pressure of the instrument. I do not, however, suffer it to be removed immediately on discovering its separation from the body of the Tibia; but generally let it remain in its situation two or three days, in order to give sufficient time for its being completely disengaged, that it may be removed with less danger of being broken. I then complete the removal in the most gentle manner; sometimes by the forceps, sometimes by passing either the one side of a pair of forceps, or a director, under it, as circumstances may dictate.* Having proceeded thus far, my attention is next directed to the removal of the loose pieces of bone within the cancelli. In almost every instance in which the process above described has been followed, I have found this bone at perfect liberty. But before I attempt its extraction, I always introduce a probe, in order to ascertain its exact position. In most of the cases which have come under my care, it is readily felt by the probe; but the practitioner should be prepared to meet with some disappointment here. There are cases, in which, from its smallness, it is not only extremely difficult to find the imprisoned piece, but even to discover any vestige of it, on the first examination. In three cases I found it so small as to require a very nice management of the probe to feel it; in two of these cases, the loose piece was found immediately after the enlargement of the aperture in the Tibia was accomplished; in the third case, it was not found till two days afterwards. Out of the whole number of cases, which have occurred in my practice, I can advert only to one in which I have searched in vain for it: nor do I feel warranted to conclude, that even in this case there was no such piece; but rather suppose, that, by its minuteness, it either eluded the search with the probe, or escaped unperceived in the dressings. This solitary instance occurred, before I was aware how very small some of these pieces are found to be. In the three cases I have mentioned, the loose pieces found would have escaped my notice, had I relaxed but a little of that perseverance with which I searched, and any conclusion against their existence, drawn from the disappointment, would, of course, have been erroneous.

"After having enlarged the aperture in the Tibia, by the caustic process, already described, and having found the loose exfoliation, I have in almost every case been able to extract it. There has, however, been an instance or two, in which it has been found still too large to pass through the aperture; although it could be moved upwards and downwards, in its bony case. In these cases I have been obliged to adopt the method already described in page 11; viz. of breaking the loose bone into two parts, in order to liberate it from its confinement. Had I not been able to do this, I must probably have been obliged to make use of the trephine.

* This exfoliation generally exhibits a very curious appearance. From an examination of seven or eight pieces of bone thus exfoliated, from as many different cases, it is evident that the ulcerative process, at the commencement of this disease, erodes the substance of the Tibia, in this part, to such a degree, as to render the enlargement of the hole, by the caustic, more certain, than it would have been, had the Tibia been of its usual thickness.

"Having carefully examined all the pieces thus extracted in the different cases which have come under my observation, I have invariably found them to be exfoliations of the interior laminæ of the Tibia; no marks of affinity with the external surface of the bone having been found on any of them. I am likewise certain that none of the exfoliated pieces extracted from within the cancelli, were any part of the exfoliation made by the caustic, for the purpose of enlarging the hole in the Tibia. The greater number of them were ascertained to be within the cavity of the bone, before any caustic was applied to the Tibia. Besides which, I should inform the reader, that the pieces exfoliated by the caustic were removed with so much care, that no fracture of them was likely to occur; and if it had, it must have been perceived.

"After I have extracted one piece of bone from within the cancelli, I examine the bottom of the wound with a probe, to ascertain whether there be any more pieces: this I continue to do occasionally for about a fortnight, knowing that no cure can be obtained if any portion of bone be left behind. The hole indeed would fill up with granulations, but a small oozing wound would remain. When the cancelli are cleared of these exfoliations, the wound should be dressed in the most superficial manner, and with the most simple dressings; applying compresses and a roller lightly over them.---In the greater number of these cases, the skin is perfectly cicatrized in the course of six weeks, or two months, from the removal of the exfoliated pieces; and the inequality on the surface of the Tibia is generally removed. The scar, indeed, and the external appearance of the leg, is somewhat different, after the cure in these cases, from what I have observed in most others. In a few instances, however, I have seen the wound continue open for several months, after the removal of the bones. In one case, indeed, it continued open nearly two years, during which time a probe could be passed within the cancelli, to the extent of four or five inches; but no material inconvenience accrued from it to the patient during this interval, and it afterwards healed up."

Under this treatment, the usual termination of the disease, with Mr. Whateley, has been in a speedy recovery. There have, however, occurred sometimes severe symptoms, which have not only endangered the limb, but have even destroyed the patient.

In two cases, in middle aged women in the intervals of child-bearing, the *infortunia* were most formidable, and the consequences extremely serious.* They are attributed by the author, to a violent use of the forceps in extracting the exfoliations from the cavity of the *Tibia;* but they are more fairly

* These were violent inflammation, attended with great pain, extending itself through almost the whole of the Tibia; and the formation of pus on the surface of its different sides, in almost every direction, as well as within the cavity of the bone. In one of these cases, the symptoms arose to such a height, as to destroy the patient in the course of a few weeks. In the other, the patient survived; but the irritation and inflammation were so great, for the first six months, as at length to leave the Tibia eroded through its entire thickness, a little referrable to the patient's walking considerable distances while under surgical treatment, and serve as a proper warning to those who may have the management of similar cases; and will induce them to confine their patients to the house, if not to the bed, during the treatment.

The remaining part of the essay consists of twenty-two cases, with a plate and explanations.

The value of this pamphlet arises from its practical utility, and as affording additional information from that best source, an observance of nature, on a disease, though not of frequent occurrence, of great importance. If we differ in some points, possibly of opinion only, with Mr. Whately, we admit in the fullest manner the service this species of medical literature is to the science; where, even if the hypothesis be wrong, the facts, if correctly observed and faithfully related, afford materials for a more legitimate theory, and more certain conclusions.

"This disease," says Mr. Whately, "differs totally from those affections of the Tibia produced by syphilis, as it does likewise from those usually denominated NECROSIS." There certainly are no facts that can induce us to suppose it has a syphilitic origin; but that it is a species or variety of the disease examined and so well described by Mr. Russell, (*Practical Essay on a certain Disease of the Bones, termed Necrosis*, 8vo. Edin. 1794), and by Professor Weidman, (*De Necrosi Ossium, fol. Francfurti ed. Mænum*, 1793), there is surely but little doubt. The disease which these writers have described in the advanced period, Mr. Whately has seen in the incipient state; or, possibly, in a mild variety, where only a small portion of bone has perished, but which being confined within a bony sheath, has kept up ulceration, until it has been removed. There are some particulars, however, in which the disease described by Mr. Whately differs from the definition of *Necrosis*, as given by Mr. Russell. In Mr. Whately's cases there seems to have been simply an exfoliation of the internal substance of the Tibia, which falling down among the cancelli and lying loose, keeps up an irritation and purulent discharge; while in the cases that Mr. Russell denominates *Necrosis*, a covering of new osseous matter surrounds the dead bone. The period of attack is likewise different. Mr. Russell found *Ne-*

little below the knee; where, on moving the limb, a kind of joint was observable. By the use of opium, fomentations, and emollient poultices, (the limb being kept in an horizontal position) the disease gradually abated; and after some large exfoliations from the substance of the Tibia had been thrown off, the use of the limb was restored.

*crosis* to be properly a disease of the early period of life, "I have never known," he says, (p. 92) "a case of it in which the attack began after the twentieth year, except in cases of *Necrosis* of the lower jaw. About the age of puberty, or from twelve to eighteen years of age, is the time of life at which patients are most liable to be attacked." Mr. Whately found that in twenty-two cases, eighteen occurred to persons past thirty. We are disposed to think, from some cases that have fallen under our own particular knowledge, as well as from an examination of the numerous *sequestra* preserved in an anatomical museum of great extent, that Mr. Russell has too much contracted the period in which this disease attacks; and that its depredations in advanced life are not confined to the lower jaw. Professor Weidman gives a plate (*Tabula tertia*) that so much resembles the plate, and so closely accords with the description given by Mr. Whately, that the identity of the diseases seems very completely established.

The mode of treatment here adopted for enlarging the opening in the Tibia, through which to extract the *sequestra*, does not quite agree with the dexterity of modern surgery: neither does it appear to us adequate to the object. We assert this, subject however, to the correction which the great experience of Mr. Whately can well supply.

---

*A Commentary on the Treatment of Ruptures, particularly in a state of Strangulation. By* E. Geoghegan. 8vo. pp. 95, London, 1810.—Highley.

To deviate from the common path of the profession with safety and success, is not the work of ordinary minds, and he may justly be considered as the benefactor to his art who shall suggest any real improvement in the treatment of a disease which has occupied the attention and the pen of so many eminent writers. The interesting little work before us has, besides its intrinsic merit, a claim to our notice, in as much as the subject was many years since opened in the pages of this Journal. In the year 1800, the author published some observations on this disease, in our 4th volume, and his farther experience has confirmed him in the validity of the opinions he then advanced. The present Commentary on the treatment of Hernia, begins with a detail of the general directions given by different authors to accomplish the reduction by the hand, and after citing the opinions of Mr. Pott, Dr. Munro, Mr. Bell, Mr. Cooper, and Mr. Lawrence, the author says, (p. 28)—

"When

When I consider the talents, and the extensive opportunities which these writers possessed, it is not without anxiety that I contest their opinions; but experience has abundantly shewn that they are ineffectual in practice, and I feel a conviction that they are unsupported by theory. I have given their own words that their full meaning may the more clearly appear, and that my inference may the more readily be understood.

The first objects for our consideration, are the structure of the parts concerned, the phenomena which the disease exhibits, and the pathology which may be fairly induced. When Hernia has taken place in the groin, it has passed through two apertures; that which is next the abdomen, is formed by the tendon of the Transversalis and internal oblique muscles, and a fascia that lines the transversalis; the external aperture is formed by a separation of the tendonous fibres of the external oblique muscles; they are filled up with the spermatic chord in males, and by the round ligaments of the uterus in females; they form an oblique canal, which in old ruptures becomes straight, or nearly so. In crural Hernia, it passes under Poupart's Ligament, through a small aperture, in a tendon formed for the transmission of the femoral vessels; during a length of time it slips in and out of the abdomen uninterruptedly, a circumstance to which I would particularly direct the attention; suddenly after any great exertion of the body, such as lifting a weight, coughing, sneezing, &c. or external injury, it refuses to return into the abdomen; on examination it appears tumified, tense, and inflamed, accompanied with excessive pain, and derangement of the functions of the viscera, and universal disturbance, the inflammation rapidly increases, the distension is prevented by the rigidity of the tendinous aperture, or by the neck of the sac which contains the intestine—hence strangulation.

The author then goes on to state, what, however, has been stated by others, but the consequences which he deduces are his own, and important; that the strangulation and its symptoms are not produced by any altered state of the abdominal aperture, and that the inflamation and enlargement of the intestines are the essential points to be considered.

He then observes,

"From what has been stated, it appears that the indication of cure generally laid down, is not warranted by the nature of the disease, nor of the treatment, namely, to return the Hernia through the apertures by pressure with the hands, because, as mentioned before, it is not the situation of the intestine, it is the enlargement of it which occasions the symptoms, and that state should, in the first instance, forbid the attempt on account of its obvious impracticability."

These remarks lead the author to, and prepare the reader for a very material alteration in the treatment of strangulated Hernia in its *first* stages, which we shall state in his own words.

"With the view of affording a satisfactory explanation of the practice which I think ought to be pursued, founded on the principles which I have endeavoured to establish, I shall describe my own manner of treating a case, and the order which I think ought to be followed, and which which has succeeded under my arrangement in, I believe, more than 36 cases since the year 1796.

I place the patient in a recumbent position, with his shoulders a little raised to relax the trunk, but the pelvis not raised, as that would put the fasciæ on the stretch ; the knees are to be drawn up, if the parts have not been irritated by handling them, or the body disturbed by jolting it about, or by any such roughness, I proceed directly to apply cloths, wet with cold water, expose the entire body naked to the air, the doors and windows being open. This practice usually succeeds within an hour,* if it does not, I surround the Hernia with my hand, or hands, at about its middle, in the way that I would grasp a gum elastic bottle, to press out its air, or other contents, by gently approximating its sides, always holding in view that the tumor is to be emptied, and not pushed up,† and that a little assistance to the compressing force, which the coats of the intestine, (re-acting from distension) are exerting, by lessening its area, even in a small degree, the air will be strongly impelled against the part of the tube which is closed, and through which it is only necessary that it should obtain exit, to effect our purpose. When it is small, as in femoral Hernia, or in protrusions, which do not pass below the groin, or are elsewhere situated, it may be done with the fingers and thumb of one hand ;—having applied the hands, I do not remove them for fifteen or twenty minutes, aware that reiterated impulses irritate, and that the effects of compression are lost each time that it is intermitted.

Cases which have been long strangulated, and those which are accompanied with great pain and tension, are exceptions to this practice, so far as the employment of the hand, on the principle that the re-action of the intestine is at a pitch sufficient to propel the air through the coalessed tube, if its passage is possible, and that handling parts in such a state would endanger mortification or rupture of them. My chief reliance in such is on bleeding, proportioning the quantity by nearly the following scale :

If the patient is young and robust, I take off sixteen ounces ; if old, or weakened by previous illness, from six to ten ounces, should even debility ‡ take place if attended with great pain, I repeat the bleeding after three hours; but if the pain yields, I omit it; all this time I apply nothing topically, except cold water, and strictly avoid all attempts with the hand.

I direct no medicine internally, and by these means I have succeeded in the instances before mentioned, and I am persuaded that they will scarcely ever fail, provided, that the taxis in the usual manner had not been practised, and that such other exasperating causes, as putting the

---

* In some cases where I could not immediately attend, I have directed that cold applications should be used until my arrival, and after an hour, they informed me that they were seized with a shivering, that they heard the wind rush out of the hernia, and that they were instantly relieved.

† I never press the hernia in any direction, or at all towards the aperture.

‡ It is very material in this, indeed in every disease, to discriminate between that debility which is the effect of pain, anxiety, and restlessness, and that which is the effect of impaired health or of original constitution; in the former, bleeding is advisable, in the latter it is generally improper to any extent.

patient patient with his head to the ground and his heels up, and jolting him about, had been omitted.

Strangulated Hernia, under this treatment, will in many cases continue several days without serious mischief, and in the event of the operation being required, peritoneal inflammation will be provided against.

The tobacco glyster is the next remedy which I would advise; in a robust subject in the proportion of one drachm to a pint of water, which I would repeat of double that strength, if the first produced no effect; in an enfeebled person, half a drachm should be first tried.

In the event of failure in our efforts to remove the strangulation, the operation of enlarging the apertures by the knife must be had recourse to; the precise time at which we should lay aside all other means, and commence this practice, is not easily decided on.

In a young and full person suffering great distress, I would not defer it longer than six or eight hours, particularly if the Hernia had been much handled, and also if bleeding had not been used; but where no mischief had been produced by external causes, and this evacuation had been premised, it might be deferred sixteen or twenty-four hours, always watching if the belly became sore to the touch, or much inflated, in which case to operate instantly. In an aged or debilitated subject it may be delayed longer. I have known it to continue in such, six, seven, or eight days, and afterwards to be removed without an operation.

Such is the treatment, and such the means which the author recommends in the strangulated Hernia previous to operation. The points on which he differs from others are, chiefly, abstaining from violent attempts to reduce the gut, and objecting to the use of purgative medicines. He is the advocate of bleeding, of the application of cold, and, in short, of every antiphlogistic plan to reduce the size and inflammation of the intestine preparatory to its return. On the whole, we consider this commentary as a valuable addition to our information on a disease of great importance, both from its frequent occurrence, and the imminent danger which accompanies it, and we therefore strongly recommend the work to our readers.

